# Conventional MRI radiomics in patients with suspected early- or pseudo-progression

**DOI:** 10.1093/noajnl/vdz019

**Published:** 2019-09-01

**Authors:** Alexandre Bani-Sadr, Omer Faruk Eker, Lise-Prune Berner, Roxana Ameli, Marc Hermier, Marc Barritault, David Meyronet, Jacques Guyotat, Emmanuel Jouanneau, Jerome Honnorat, François Ducray, Yves Berthezene

**Affiliations:** 1 Department of Neuroradiology, East Group Hospital, Hospices Civils de Lyon, Lyon Cedex, France; 2 Department of Molecular Biology, East Group Hospital, Hospices Civils de Lyon, Lyon Cedex, France; 3 Department of Neuropathology, East Group Hospital, Hospices Civils de Lyon, Lyon Cedex, France; 4 Department of Neurosurgery, East Group Hospital, Hospices Civils de Lyon, Lyon Cedex, France; 5 Université Claude Bernard Lyon 1, Villeurbanne, France; 6 Department of Neuro-Oncology, East Group Hospital, Hospices Civils de Lyon, Lyon Cedex, France

**Keywords:** artificial intelligency, deep learning, magnetic resonance imaging, glioblastoma, pseudoprogression

## Abstract

**Background:**

After radiochemotherapy, 30% of patients with early worsening MRI experience pseudoprogression (Psp) which is not distinguishable from early progression (EP). We aimed to assess the diagnostic value of radiomics in patients with suspected EP or Psp.

**Methods:**

Radiomics features (RF) of 76 patients (53 EP and 23 Psp) retrospectively identified were extracted from conventional MRI based on four volumes-of-interest. Subjects were randomly assigned into training and validation groups. Classification model (EP versus Psp) consisted of a random forest algorithm after univariate filtering. Overall (OS) and progression-free survivals (PFS) were predicted using a semi-supervised principal component analysis, and forecasts were evaluated using C-index and integrated Brier scores (IBS).

**Results:**

Using 11 RFs, radiomics classified patients with 75.0% and 76.0% accuracy, 81.6% and 94.1% sensitivity, 50.0% and 37.5% specificity, respectively, in training and validation phases. Addition of *MGMT* promoter status improved accuracy to 83% and 79.2%, and specificity to 63.6% and 75%. OS model included 14 RFs and stratified low- and high-risk patients both in the training (hazard ratio [HR], 3.63; *P* = .002) and the validation (HR, 3.76; *P* = .001) phases. Similarly, PFS model stratified patients during training (HR, 2.58; *P* = .005) and validation (HR, 3.58; *P* = .004) phases using 5 RF. OS and PFS forecasts had C-index of 0.65 and 0.69, and IBS of 0.122 and 0.147, respectively.

**Conclusions:**

Conventional MRI radiomics has promising diagnostic value, especially when combined with *MGMT* promoter status, but with moderate specificity. In addition, our results suggest a potential for predicting OS and PFS.

**Key Points**

Conventional MRI radiomics has interesting diagnostic value in the diagnosis of pseudoprogression but its specificity remains moderate. It may have a substantial interest in forecasting overall and progression-free-survivals.

**Importance of the Study**

Within 6 months after the end of concomitant radiochemotherapy, up to 30% of glioblastoma patients have an increase or new enhancing lesions on MRI, not resulting from early progression (EP) but from radiation-induced changes which are called pseudo-progression (Psp). This phenomenon remains a daily problem because no diagnostic method has yet been validated. Radiomics is an emerging technique presupposes that the quantification of certain image properties allows a better characterization of the tumor phenotype and its microenvironment. Studies have reported its ability to predict overall survival (OS) in treatment-naive patients, and OS and progression-free survival (PFS) in second-line treatment patients. Our study aimed to assess the value of conventional MRI radiomics in patients with suspected EP and Psp. Our results show that radiomics has promising diagnostic value, especially when combined with *MGMT* promoter status, but its specificity is moderate. In addition, our study suggests that this method may forecast OS and PFS in these patients.

Glioblastomas (GBM) account for the majority of malignant brain tumors in adults. Standard first-line treatment usually consists of concurrent radiochemotherapy (RCT) and adjuvant temozolomide (TMZ) therapy.^1^ During the first 6 months of follow-up, nearly 20% to 30% of patients experience treatment-related subacute reactions, resulting in increased or new lesions.^2,3^ Since these reactions mimic an early progression (EP), they have been called pseudo-progression (Psp). Although some signs may suggest EP rather than Psp, there are no criteria for a diagnosis of certainty using conventional MRI.^4,5^ To date, only a new histological examination can characterize a worsening of the MRI within 6 months following radiochemotherapy.^6^ In clinical practice, a second surgery is difficult to accept and histological characterization can also be complicated.^6^ Therefore, the current recommendation in cases of suspected Psp is to continue TMZ and to conclude after a follow-up MRI.^6^ In case of regression or stabilization of enhancing lesions, the diagnosis of Psp is retained while an increase establishes EP.^6,7^ Thus, the final diagnosis in these patients can only be made retrospectively. This has important implication for individual care and for clinical trials since it is recommended to exclude these patients from trials.^7^ Although advanced MRI techniques such as diffusion-weighted-imaging, dynamic-susceptibility-contrast imaging, and spectroscopy have shown promising diagnostic performances, acquisition protocol for evaluating glioblastomas is variable among imaging centers and mainly relies on conventional MRI.^8^ In addition, only two-dimensional measurements based on conventional MRI sequences are currently validated for GBM follow-up.^9,10^

Recent advances in high-resolution image acquisition, computational hardware, and high-dimensional data processing allowed quantifying innumerable texture and shape characteristics of medical images.^11^ This broad set of methods have been called radiomics.^12^ It presupposes that the quantification of certain image properties, such as variations in gray levels, allows a better characterization of the tumor phenotype and its microenvironment.^11^ In neuro-oncology, this method has demonstrated its ability in predicting survival of newly diagnosed GBM patients and to determine the status of the *MGMT* promoter methylation and *Isocitrates Dehydrogenases* mutation.^13–16^

This study aimed to assess the diagnostic value of conventional MRI radiomics in the management of patients with suspected EP or Psp. Our primary objective was to evaluate the diagnostic performance of radiomics alone or in combination with *MGMT* promoter status. Our secondary objective was to study the ability to predict overall survival (OS) and progression-free survival (PFS).

## Materials and Methods

### Patient Selection, Collection of Survival Data and of the MGMT Promoter Status

Institutional Review Board approved this retrospective study, and the requirement for written consent was waived.

Patients were identified in our institution’s radiological database. As previously proposed,^4^ they were included if they had received a histological diagnosis of GBM according to WHO 2016, if they were over 18 years of age, if they had received TMZ radiochemotherapy as initial treatment, if they had radiological and clinical surveillance data available, if they had experienced a radiological progression within 6 months of their concomitant TMZ RCT, and there were no significant MRI artifacts. In line with RANO criteria, radiological progression was defined as a significant increase of at least 25% in enhancing lesions or any new lesion.^10^

The final diagnosis was established by consensus by a neuro-oncologist and a radiologist. EP and Psp were diagnosed on the basis of histological analysis or, in the absence of histological proof, by radioclinical follow-up. EP was retained if follow-up MRI demonstrated a radiological progression defined according to RANO criteria. Psp was established based on follow-up MRI if there was a stabilization or a decrease in enhancing lesions without a change in treatment.

OS and PFS data were extracted from medical files. OS was determined based on the date of death or last visit, and PFS was established based on the date of the first MRI that objectified a radiological progression followed by further therapeutic adjustment.

The determination of *MGMT* promoter methylation status was obtained with the methylation specific polymerase reaction using the surgical piece from the first surgery, with methods described previously.^17^

### MRI Characteristics and Radiomics Features Extraction

Images were acquired using either 1.5 Tesla (Siemens, Avento) or 3 Tesla (Philips Achieva, Philips Healthcare). All studies included axial pre-contrast fluid attenuation inversion recovery (FLAIR) images and 3D post-contrast T1-weighted images (T1CE) using a 0.1 mmol/kg dose of gadoterate meglumine (DOTAREM, Guerbet) for both magnets. For 3 Tesla MRI scan, axial FLAIR images were acquired with TI = 2,400 milliseconds, TE = 85 milliseconds, TR =8,500 milliseconds, section thickness = 5 mm, an interslice gap of 5%, and 3D T1-weighted images were acquired with TI = 1,100 milliseconds, TE = 4 milliseconds, TR = 1,710 milliseconds, FA = 15°, and section thickness of 1 mm. For 1.5 Tesla MRI scan, axial FLAIR images were acquired with TI = 2,500 milliseconds, TE = 120 milliseconds, TR = 10,000 milliseconds, section thickness = 5 mm, an interslice gap of 5%, and 3D T1-weighted images were acquired with TI = 1,100 milliseconds, TE = 4 milliseconds, TR = 8.6 milliseconds, FA = 8°, andsection thickness of 1.2 mm.

**Figure 1.**
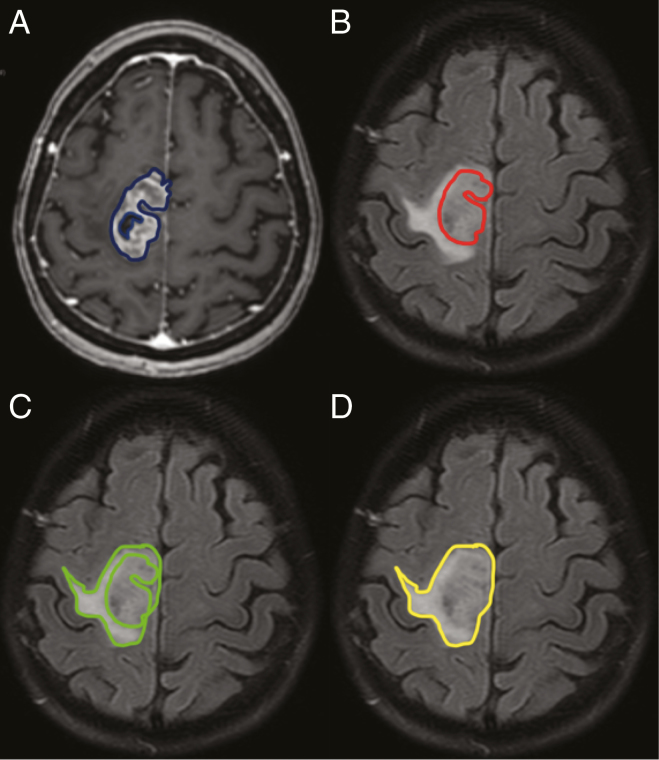
Example of MRI segmentation. Segmentation of the contrast-enhanced tumoral portion using T1-weighted contrast-enhanced images (A–blue color) and fluid-attenuated-inversion-recovery (FLAIR) images (B–red color); of the peri-tumoral parenchymal area using FLAIR (C–red color); and of a volume including the contrast-enhanced tumoral portion and the peri-tumoral parenchymal area (D–yellow color).

Radiomics features were obtained using a dedicated software, LifeX (Commissariat de l’Energie Atomique, Saclay, France).^18^ Each slice of MRI was manually delimited by an expert reader, without coregistration, to provide four volumes: (i) a volume including only the contrast-enhanced tumoral portion but avoiding large cysts obtained using T1CE images (volume T1CE); (ii) a volume contrast-enhanced tumoral portion using FLAIR images (volume FT); (iii) a volume including only peritumoral parenchymal area using FLAIR images (volume FO); and (iv) a volume including the contrast-enhanced tumoral portion and peritumoral parenchymal area using FLAIR images (volume FOT). An example of the segmentation performed is shown in Figure 1.

Thirty-nine radiomics features were extracted by volume, namely, 156 per patient. Subsequently, they were normalized to provide *z*-scores.

### Statistical Analyses and Construction of Diagnostic and Prognostic Models

Statistical computing and models’ construction were made using R version 3.2.3 (R Core TeamVienna, Austria).

### Classification Model (EP Versus Psp)

A binary classification model (EP versus Psp) was constructed using a statistical method previously described as the most robust and efficient for this type of model.^19^ Patients were randomly assigned to a training and validation group in a 2:1 ratio while maintaining EP/Psp ratio comparable to that of the overall population. First, the radiomics features were selected according to their importance according to a Wilcoxon-test–based method and were further integrated into a random forest classifier. This model, which only had radiomics features, was compared with a model with only the status of the *MGMT* promoter and to a model combining both.

This step required the “caret” and “randomForest” packages implemented in the R software using the default settings as suggested by the authors.^20,21^

### Survival Models (OS and PFS Forecasts)

Patients were divided into training and validation groups in a 2:1 ratio, with balanced survival between the two groups. Subsequently, a supervised principal component analysis (SPCA) was conducted on the training group data to identify the most predictive radiomics features. This analysis was then applied to the data of the validation group. The SCPA selects the parameters most associated with the outcome. Articles have reported the robustness and performance of this method for quantitative outcomes, particularly in survival models.^22–25^ In addition, this method has already been used in previous articles which aimed to determine the prognostic value of radiomics analyses in newly diagnosed and recurrent GBM.^13,26^ Cox regression coefficients were computed for each radiomics feature from the training data. Principal components were then calculated on characteristics whose Cox scores exceeded a threshold value, estimated by 10-fold cross-validation. Importance scores for the selected radiomics parameters were determined based on their correlation with the first SPCA. For each patient, a continuous and discrete risk score (high or low) was calculated based on this analysis. SCPA’s performance in stratifying survival (low or risk) was evaluated by Cox regression analyses. This method was used to establish two survival models: one for OS and the other for PFS.

Accuracies of these models were assessed by calculating the integrated Brier Score (IBS) which reflects prediction errors over time and the concordance index (C-index). IBS ranges from 0 for a perfect model to 0.25 for a noninformative model with 50% incidence of the outcome. The “superpc” package was used to build survival models whilst IBS was computed with “pec” package using the default settings.^23,27^

## Results

### Demographics of Included Patient

From January 2005 to July 2016, we retrospectively identified 168 GBM patients who had been treated with TMZ radiochemotherapy and for whom radiological follow-up was available. Of these patients, 105 showed radiological progression within 6 months after completing TMZ radiochemotherapy. Twenty-nine patients were excluded from the radiomics analysis due to MRI artifacts (*n* = 7) and uncertain diagnosis (*n* = 22). Fifty-three patients (59.3%) were diagnosed with EP, 49 by follow-up and 4 by a new histological examination. Twenty-three (30.7%) patients were diagnosed with Psp, all based on follow-up. The demographic and clinical characteristics of the 76 patients included are presented in Table 1.

**Table 1.** Patient demographics

	Pseudo-progression	Early Progression	*P*
Age (year)	54.9 ± 1S2.7	59.2 ± 9.5	0.11*
Sex			0.453**
Female	11 (47.8%)	20 (37.7%)	
Male	12 (52.2%)	33 (62.3%)	
Extent of surgery			
Biopsy	9 (39.2%)	25 (47.2%)	0.618*
Subtotal resection	7 (30.4%)	10 (18.9%)	0.368*
Gross total resection	7 (30.4%)	18 (33.9%)	>0.99*

*Calculated using two-tailed Fischer’s exact test.

**Calculated using two-tailed Student test.

The status of the *MGMT* promoter was available in 71/76 patients, including 49 EP (14 patients with a methylated *MGMT* promoter) and 22 Psp (18 patients with a methylated *MGMT* promoter).

**Figure 2.**
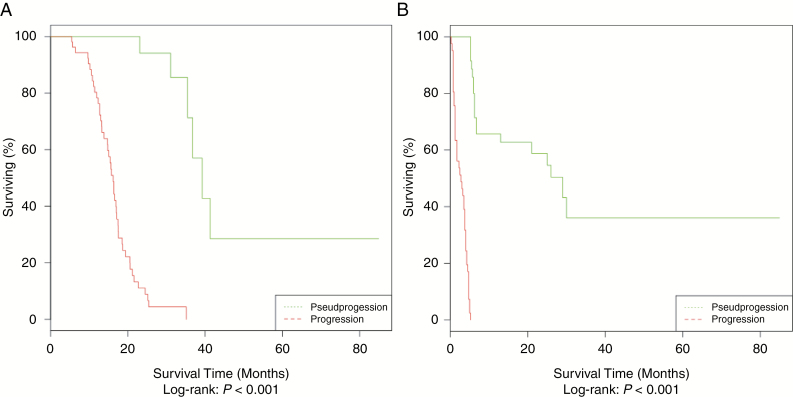
Kaplan–Meier plots of overall- and progression-free-survival of included population. Kaplan–Meier plots show overall survival (A) and progression-free-survival (B) according to the pseudo- or early-progression diagnosis.

The median OS was significantly longer in the Psp group with an OS of 39.3 months (95% CI [35.4-NA]) versus 16.2 months (95% CI [14.7–17.4]) in the EP group. The median PFS was not calculable in the Psp group (95% CI [29.0-NA]) and was 3.8 months (95% CI [2.8–4.8]) in the EP group. The Kaplan–Meier plots of OS and PFS are presented in Figure 2.

### Classification Model (EP Versus Psp)

Using the 52 subjects of the training group, 11 radiomics features were selected based on Wilcoxon-test–based method and integrated into a random forest classifier. These parameters, their scores, and mean values in Psp and EP subjects are presented in Supplementary Table 2.

For the model including only radiomics features, accuracy, sensitivity, and specificity were 75.0% (95% CI [60.4–86.4]), 81.6%; 95% CI [65.7–92.3], 50.0%, 95% CI [18.7–81.3], respectively, in the training set, and 76.0% (95% CI [54.9–90.6]), 94.1% (95% CI [71.3–99.8]), 37.5% (95% CI [8.5–75.5%]) in the validation set.

**Figure 3.**
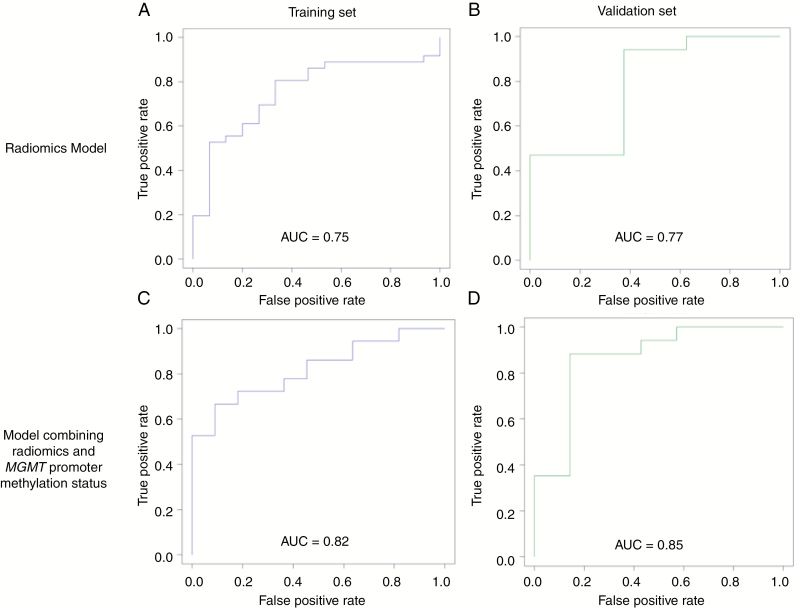
Receiver-Operating Characteristic curves of the binary classification model. This figure shows Receiver-Operating Characteristic curves of the radiomics model in the training group (A), and in the validation group (B), of the *MGMT* promoter status model (C and D), and of the combination of radiomics and *MGMT* promoter status models.

The combination of radiomics and *MGMT* promoter methylation status improved diagnostic performance with an accuracy of 83.0% (95% CI [69.2–92.4]), a sensitivity of 88.9% (95% CI [73.9–96.9]), and a specificity of 63.6% (95% CI [29.9–80.3]) in the training set. During the validation phase, accuracy, sensitivity, and specificity were 79.2% (95% CI [59.9–92.9]), 80.0% (95% CI [56.3–94.3]), and 75.0% (95% CI [19.4–99.3]), respectively. The receiver operating characteristic curves of both models are found in Figure 3.

### Survival Models (OS and PFS Forecasts)

**Figure 4.**
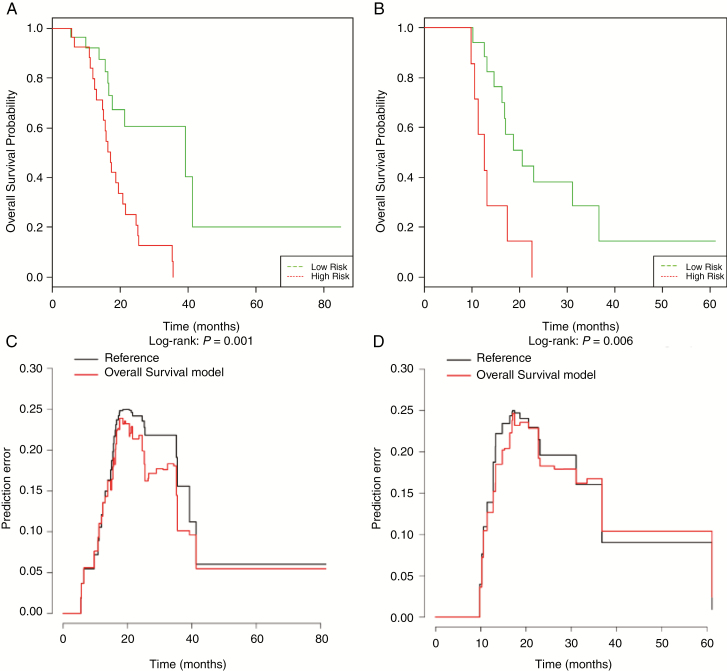
Performance of the overall-survival prediction model. Kaplan–Meier plots show overall-survival (OS) for patients in the training group (A) and in the validation group (B), stratified to low- or high-risk group according to the semi-supervised principal component analysis model. The prediction error curves show the forecasts of the OS model (in red) compared with the observed data (in black) in the training group (C) and in the validation group (D).

Using the 52 subjects in the training group, SCPA identified 14 radiomics features that were the most important predictors of OS using a 10-fold cross-validated threshold. These radiomics features, their importance scores, and their normalized values (*z*-scores) are presented in Supplementary Table 3. Subsequently, they were integrated to generate continuous and dichotomous SCPA risk scores to identify patients with low or high risk of mortality. The median OS was 15.6 months (95% CI [13.8–17]) for the high-risk group versus 35.4 months (95% CI [31.1-NA]) for the low-risk group (*P* < .001). In the training set, this score stratified patients into low- and high-risk groups with a risk ratio (HR) of 3.63 (95% CI [1.62–8.2], *P* = .002), a C-index of 0.63, and an IBS of 0.092. Forecast accuracy was comparable in the validation set, with a HR of 3.76 (95% CI [1.37–10.2], *P* = .001), a C-index of 0.65, and an IBS of 0.122. The OS of low-risk patients were significantly longer in the training and in the validation groups (Log-rank: *P* < .001 and *P* = .006). The Kaplan–Meier plots and prediction error curves of both groups are found in Figure 4.

**Figure 5.**
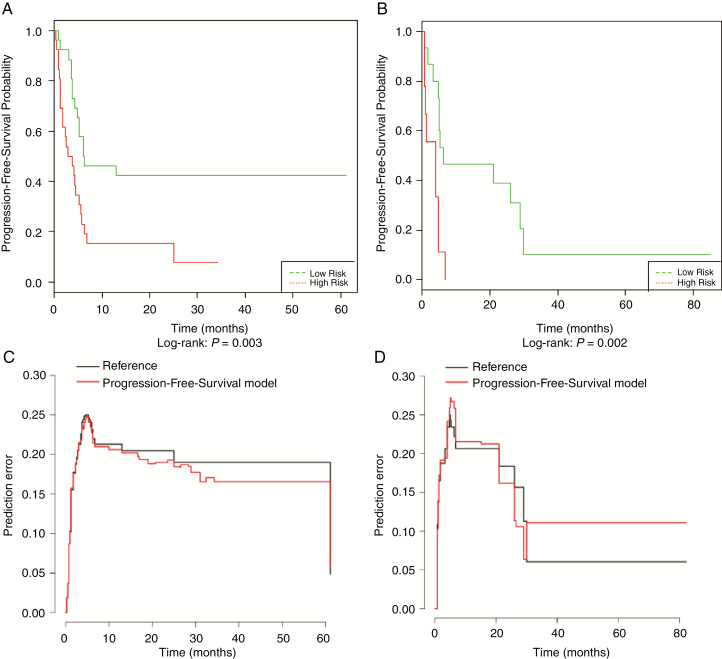
Performance of the progression-free-survival prediction model. Kaplan–Meier plots show progression-free-survival (PFS) for patients in the training group (A) and in the validation group (B), stratified to low- or high-risk group according to the semi-supervised principal component analysis model. The prediction error curves show the forecasts of the PFS model (in red) compared with the observed data (in black) in the training group (C) and in the validation group (D).

Similarly, five major radiomics predictive features PFS were selected by SCPA model using a 10-fold cross-validation threshold. These radiomics features, their importance scores, and their standardized values (*z*-scores) are presented in Supplementary Table 4. As above, continuous and dichotomous SCPA progression risk scores were generated. The median PFS was 2.8 months (95%CI [1.8–3.8]) for the high-risk group versus 29.0 months (95% CI [13.1-NA]) for the low-risk group (*P* < .001). In the training set, this score dichotomized patients into low- and high-risk groups with a HR of 2.58 (95% CI [1.34–4.99], *P* = .005), a C-index of 0.63, and an IBS of 0.147. During the validation phase, this score separated high- and low-risk patients with a HR of 3.58 (95% CI [1.61–11.9], *P* = .004), a C-index of 0.69, and an IBS of 0.157. The Kaplan–Meier plots and prediction error curves of both groups are found in Figure 5.

## Discussion

This study aimed to evaluate the diagnostic performances of conventional MRI radiomics in patients with suspected EP or Psp. Our results suggest that this method has interesting diagnostic performances, especially when combined with the analysis of MGMT promoter status but its specificity is moderate.

In agreement with previous reports, the AUCs for radiomics analysis alone were 0.75 in the training phase and 0.77 in the validation phase compared with the reported AUC ranging from 0.74 to 0.86.^28–30^ However, the specificity of the radiomics model was low, ranging from 50.0% in the training set to 37.5% in the validation set, whereas X. Chen et al. reported a specificity of 100% with contrast and correlation derived from gray-level co-occurrence matrix on T2 weighted-images,^28^ and J.Y. Kim et al. a specificity of 78.1%.^31^ The reasons for this difference are unclear and might be related to different recruitment and classification of patients. For example, X. Chen et al. included 12 patients classified as Psp, all of whom had histological evidence of radiation necrosis, and J.Y. Kim et al. defined Psp both using histological evidences (*n* = 4) and clinical follow-up (*n* = 42). This demonstrates a confusion between Psp and radiation necrosis in the literature.^30^ Narrowly, Psp is a clinical diagnosis defined by favorable evolution of enhancing lesions at follow-up while radiation necrosis is a histological diagnosis.^30^ Although some have postulated that Psp and radiation necrosis exist at different time points,^32^ this dichotomization is being questioned.^30^ Indeed, it has been reported that the occurrence of radiation necrosis is not uncommon within 6 months after radiotherapy.^33^ Consequently, the 6-month threshold separating Psp from radiation necrosis that we have used and as used in most of studies might be arbitrary, leading to the inclusion of heterogeneous population. Indeed, some authors consider that these two entities lie along a spectrum of post-treatment radiation effects and are not definable one from each other.^30^ Since post-treatment radiation effects include acute, subacute, and delayed reactions that depend on different pathogenic mechanisms,^34^ there is no evidence that these reactions share the same phenotype. Finally, there is nothing to formally exclude the possibility that patients experience imaging worsening related to both tumor recurrence and post-treatment radiation effects when the final diagnosis is established by the follow-up, especially since these patients are undergoing TMZ treatment.

The recognition of Psp is important to provide optimal patient care, but the criteria for defining Psp vary in the literature. In this context, our secondary objective was to assess the value of conventional MRI radiomics in predicting survivals. Our results are likely to suggest that this method is promising in the prediction of OS and PFS. Using a relatively limited number of radiomics features, survival models stratified patients into high and low risk for recurrence and/or mortality. Consistent with previous studies, all of the selected radiomics features indicated greater image heterogeneity in patients with worse prognosis.^28,29,31^ Eleven radiomics features out of 14 selected for the OS prediction model and all those selected for the PFS prediction model were extracted from the FLAIR sequences. Most of them were derived from volumes including peritumoral parenchymal area, 8/14 in the OS model, and 4/5 in the PFS model. These results are in line with previous studies that reported that GBM heterogeneity involved peritumoral parenchymal area^35,36^ and that nearly 90% of GBM recurrence occurred in this area.^37^ In a previous study, Kickingereder et al. reported that the radiomics-based classification of recurrent glioblastomas predicted survival and stratified patients receiving second-line antiangiogenic therapy into high- or low-risk groups for recurrence. They concluded that this classification might help therapeutic decisions by selecting patients who may potentially gain benefit from antiangiogenic therapy. Likewise, our secondary results suggest that the radiomics-based stratification of patients with early imaging worsening into high- and low-risk groups for recurrence and/or mortality may be a valuable imaging biomarker in the monitoring of therapeutic response in first-line treatment.

This study has certain limitations. Other than its retrospective and its mono-centric nature the main limitation of this study is the absence of an external validation cohort. As a consequence, our results present a risk of overfitting of the data and will need to be validated in an independent series. In addition, radiomics analysis was restricted to conventional MRI data and advanced MRI methods such as diffusion-weighted-imaging and dynamic-susceptibility-contrast imaging have not been assessed. The tumor subregions were defined manually, which may be less reproducible than algorithmic methods, but appeared to us to be closer to clinical practice. Survival data were considered in the final diagnosis and radiomics features were only measured at the time of the MRI showing a worsening of enhancing lesions. It has been reported that the radiomics phenotype of naïve-treatment GBM was correlated with survival outcomes.^13–15^ As a consequence, we cannot exclude that our results are related to the original GBM phenotype and patients’ selection. In addition, the available sample size was small and markedly limited our radiomics-based survival analysis. Prospective and multi-institutional studies evaluating the evolution of radiomics features under treatment are needed to establish the clinical relevance of this method in the evaluation of treatment response. These studies should analyze conventional and advanced MRI features together with GBM molecular characteristics that may affect treatment response (i.e., MGMT methylation, IDH mutation, and TERT promoter mutation status) and include an external validation cohort. Regarding survival prediction based on post-treatment radiomics, it would be interesting to assess whether this approach can be successfully applied to the first MRI performed after concomitant radiochemotherapy completion irrespective of the suspicion of Psp or EP.

In summary, our study shows that conventional MRI radiomics has promising diagnostic value, especially when combined with *MGMT* promoter status, but its specificity is moderate. In addition, our results suggest that this method may forecast OS and PFS in these patients. These results however will need to be validated in an independent series.

## Supplementary Material

vdz019_suppl_Supplementary_Table
