# Supplementary material for: Conventional MRI radiomics in patients with suspected early- or pseudo-progression
Source: Neurooncol Adv. 2019 Sep 1;1(1):vdz019. doi: 10.1093/noajnl/vdz019 (PMC7212855; doi:10.1093/noajnl/vdz019)
Supplement: vdz019_suppl_Supplementary_Table [file vdz019_suppl_supplementary_table.docx]

**SUPPLEMENTARY MATERIAL.**

**Table 2. Ranking and z-scores of the 11 radiomics features selected in the radiomics classification model (early versus pseudoprogression).**

|  |  | Normalized z-scores* | |
| --- | --- | --- | --- |
| Textural Indices | Ranking | Pseudoprogression | Early Progression |
| GLCM Correlation (T1CE) | 1 | 1.42 ± 0.19 | 0.92 ± 0.68 |
| GLRLM HGRE (T1CE) | 2 | 2.43 ± 2.07 | 1.90 ± 2.22 |
| NGLDM Busyness (FT) | 3 | 0.02 ± 0.04 | 0.03 ± 0.07 |
| GLCM Correlation (FT) | 4 | 0.93 ± 0.04 | 0.86 ± 0.10 |
| GLZLM ZP (FT) | 5 | 0.72 ± 0.07 | 0.78 ± 1.31 |
| NGLDM Coarseness (FT) | 6 | 0.03 ± 0.02 | 0.02 ± 0.01 |
| GLZLM GLNU (T1CE) | 7 | 2.11 ± 1.72 | 2.13 ± 2.39 |
| NGLDM Contrast (FT) | 8 | 0.21 ± 0.14 | 0.33 ± 0.29 |
| GLRLM RP (FT) | 9 | 0.92 ± 0.02 | 0.94 ± 0.03 |
| NGLDM Coarseness (F0) | 10 | 0.19 ± 0.86 | 0.01 ± 0.01 |
| GLRLM LRLGE (T1CE) | 11 | - 0.84 ± 0.19 | - 0.77 ± 0.28 |

Note: Negative values are associated with worse survival while, positives values indicate better survival.  * Data are means ± standard deviations.

Abbreviations: GLCM = gray-level co-occurrence matrix; GLRLM = gray-level run-length matrix; NGLDM= neighboring grey-level matrix; GLZLM: Grey-Level Zone Length Matrix; ZP= Zone Percentage; GLNU= Grey-Level Non-Uniformity; RP= Runs percentage; LRLGE= Long-Run Low Gray-Level; T1CE = contrast-enhanced tumoral portion volume in T1-weighted contrast-enhanced images; FO= peri-tumoral parenchymal area volume in FLAIR images; FT= contrast-enhanced tumoral portion volume in FLAIR images, FOT= contrast-enhanced tumoral portion and peri-tumoral parenchymal area volume in FLAIR images,

**Table 3. Ranking and z-scores of the 14 radiomics features selected in the Overall-Survival Model.**

|  |  | Normalized z-scores* | |
| --- | --- | --- | --- |
| Textural Indices | Ranking | Low Risk | High Risk |
| GLCM Correlation (FO) | 1 | 0.40 ± 0.46 | - 0.17 ± 1.13 |
| GLCM Correlation (FOT) | 2 | 0.31 ± 0.49 | - 0.13 ± 1.06 |
| GLCM Correlation (FT) | 3 | 0.21 ± 0.77 | - 0.08 ± 1.08 |
| NGLDM Busyness (T1CE) | 4 | - 0.47 ± 0.79 | 0.20 ± 1.02 |
| NGLDM Busyness (FOT) | 5 | - 0.43 ± 0.67 | 0.18 ± 1.06 |
| NGLDM Busyness (FO) | 6 | - 0.33 ± 0.40 | 0.13 ± 1.14 |
| GLCM Contrast (FOT) | 7 | - 0.29 ± 0.60 | 0.12 ± 1.08 |
| HISTO Entropy (FO) | 8 | 0.29 ± 1.00 | - 0.12 ± 0.98 |
| GLCM Dissimilarity (FOT) | 9 | - 0.27± 0.79 | 0.11± 1.06 |
| HISTO Kurtosis (FT) | 10 | - 0.16± 0.90 | 0.07± 1.04 |
| GLRLM LRLGE (T1CE) | 11 | 0.43 ± 1.18 | - 0.18 ± 0.86 |
| NGLDM Coarseness (T1CE) | 12 | 0.43 ± 1.40 | - 0.17 ± 0.72 |
| GLCM Dissimilarity (FO) | 13 | - 0.15± 0.87 | 0.06 ± 1.05 |
| NGLDM Coarseness (FT) | 14 | 0.42 ± 1.24 | - 0.17 ± 0.83 |

Note: Negative values of importance scores are associated with worse survival while, positives values indicate better survival.  * Data are means ± standard deviations.

Abbreviations: GLCM = Gray-Level Co-Occurrence Matrix; GLRLM = Gray-Level Run-Length Matrix; NGLDM= Neighboring Gray-Level Matrix; HISTO= histograms; T1CE = contrast-enhanced tumoral portion volume in T1-weighted contrast-enhanced images; FO= peri-tumoral parenchymal area volume in FLAIR images; FT= contrast-enhanced tumoral portion volume in FLAIR images, FOT= contrast-enhanced tumoral portion and peri-tumoral parenchymal area volume in FLAIR images,

**Table 4. Ranking and z-scores of the 5 radiomics features selected in the progression-free survival model.**

|  |  | Normalized z-scores* | |
| --- | --- | --- | --- |
| Textural Indices | Ranking | Low Risk | High Risk |
| GLZLM_SZLGE (FO) | 1 | - 0.24 ± 0.31 | 0.15 ± 1.44 |
| NGLDM_Busyness (FO) | 2 | - 0.36 ± 0.39 | 0.30 ± 1.35 |
| GLCM_Correlation (FO) | 3 | 0.60 ± 0.33 | - 0.31 ± 1.10 |
| GLRLM_GLNU (FOT) | 4 | 0.06 ± 0.88 | - 0.06 ± 1.01 |
| GLCM_Correlation (FT) | 5 | 0.55 ± 0.41 | - 0.27 ± 1.11 |

Note: Negative values of importance score are associated with worse survival while, positives values indicate better survival. * Data are means ± standard deviations.

Abbreviations: GLCM = Gray-Level Co-Occurrence Matrix; GLRLM = Gray-Level Run-Length Matrix; NGLDM= Neighboring Gray-level matrix; FO= peri-tumoral parenchymal area volume in FLAIR images; FT= contrast-enhanced tumoral portion volume in FLAIR images, FOT= contrast-enhanced tumoral portion and peri-tumoral parenchymal area volume in FLAIR images,
